# Molecular Epidemiology of Leptospirosis in the Amazon

**DOI:** 10.1371/journal.pmed.0030302

**Published:** 2006-08-22

**Authors:** David A Haake

## Abstract

Haake discusses a new study that applied molecular approaches to compare the density and diversity of leptospires in urban versus rural environmental water sources in the Peruvian Amazon.

Leptospirosis is a zoonotic infectious disease that is transmitted from animals to humans primarily via water [1]. The causative agent is a bacterial spirochete that colonizes the renal tubules of infected animals and is shed in the urine for varying periods of time, depending on whether the animal is an accidental or reservoir host for that serovar (see Glossary). Rats are reservoir hosts for serovars of *Leptospira interrogans* (Figure 1) belonging to the Icterohaemorrhagiae serogroup, and infected rats probably remain infected, and infectious, for life [2]. A total of 17 species of *Leptospira* and hundreds of serovars have been described [3]. All leptospires are well suited to life in water. In fact, several leptospiral species, such as *L. biflexa*, are saprophytic organisms that are exclusively aquatic. However, even pathogenic species can survive for months in a nutrient-poor aqueous environment while waiting to encounter a new host [4].

**Figure 1:**
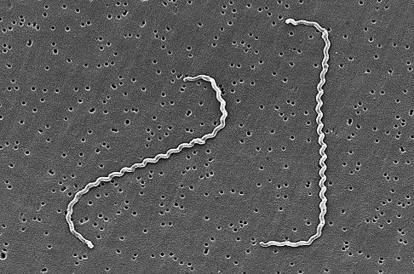
Scanning Electron Micrograph of *Leptospira interrogans* (Strain RGA) The image shows two spirochetes bound to a 0.2-μm filter. Strain RGA was isolated in 1915 by Uhlenhuth and Fromme from the blood of a soldier in Belgium. (Image: CDC/NCID/HIP/Janice Carr)

In tropical regions of the world, leptospirosis is a widespread public health problem [5]. The disease is endemic wherever open sewers or agricultural practices lead to contamination of water with animal urine. Although most human leptospirosis infections are self-limited, complications are common, involving hepatorenal failure, pulmonary hemorrhage, and death in 10%–50% of severe cases [6]. Large outbreaks of leptospirosis occur when heavy rainfall and inadequate civil engineering result in urban flooding [7]. Rat-borne *L. interrogans* species are typically isolated from patients in urban settings, but—until the publication today of a study by Vinetz and colleagues in *PLoS Medicine* [8]—little was known about the species present in environmental water sources in either urban or rural settings. Given the laborious nature of leptospiral isolation and culture techniques, particularly from environmental samples, there was previously no systematic method available to determine the identity, spectrum, and density of leptospires in water samples.

## The New Molecular Epidemiological Study

Vinetz and colleagues set out to apply molecular approaches to systematically compare the density and diversity of leptospires in urban versus rural environmental water sources in the Peruvian Amazon. Two settings were selected in and around the city of Iquitos. The first setting was Belen, an urban slum area with open sewers and frequent sightings of rats. The second area was Padrecocha, a poor agricultural area with domestic livestock and dogs.

Water sources were sampled and tested by quantitative polymerase chain reaction using a set of primers designed to target the 16S rRNA gene of leptospiral pathogens and avoid strictly saprophytic species. Not surprisingly, the highest densities of leptospires were found in open gutters in the urban area. However, high numbers (more than 2,000 bacteria per milliliter) were also found in river water in the urban area, indicating that the river was heavily contaminated with rat urine. Leptospiral DNA was also found in water samples from the rural area, but at significantly lower levels than in the urban water samples.

Amplified leptospiral 16S genes were cloned and sequenced from a number of the water samples in Belen and Padrecocha. The sequences from environmental clones were compared with sequences from human isolates obtained from patients living in urban and rural areas. Phylogenetic analysis of leptospiral 16S sequences was known to correlate well with leptospiral speciation by DNA–DNA hybridization [9]. Urban water clone sequences from Belen reflected the full diversity of leptospiral species, including known pathogens, organisms with intermediate pathogenicity, and saprophytic species. Rural water clones from Padrecocha included intermediate sequences, but none consistent with pathogens or saprophytes. Surprisingly, both the urban and rural water clones included sequences segregating to a major new branch of the leptospiral phylogenetic tree, referred to as clade C, that was previously unknown.

## An Unexpected Discovery

The discovery of a new branch of the leptospiral phylogenetic tree in the Peruvian Amazon demonstrates the power of the molecular epidemiological approach. Even though hundreds of leptospiral strains have been isolated from many sources, including South American sources, and 16S sequences are known for many of these strains, it appears that clade C sequences represent one or more species that are entirely new to research. The new leptospire(s) appears to be relatively prevalent in Padrecocha, as 13 out of 17 sequences from rural water samples belong to clade C.

A limitation of this approach is that the relevance of the new leptospire(s) to human disease is unknown. However, the molecular epidemiological approach is an important first step, alerting us to the presence of a new branch of the leptospiral phylogenetic tree, and directing us where to go to isolate the new organism(s), so that we can perform virulence testing and other fundamental types of characterization.

## Policy Implications

An important advantage of the molecular epidemiological approach is the ability to quantitate leptospiral pathogens in environmental samples. This approach is therefore an important new weapon in the public health armamentarium. Detection of significant numbers of leptospires in an urban environment should prompt education about the importance of simple prevention strategies, such as use of footwear (going without shoes is a risk factor for acquiring leptospirosis [10]) and chlorination of drinking water, to reduce the risk of exposure [11]. Another positive outcome would be to alert health-care providers in endemic regions to have a lower threshold for early antibiotic treatment of febrile illnesses in patients who have other signs and symptoms consistent with leptospirosis. In urban settings, garbage collection and other common-sense rodent-abatement strategies could be effective in reducing the density of organisms in the environment. In rural settings, improvements in animal husbandry, such as antibiotic treatment of infected animals and routine vaccination, could reduce leptospiral carriage and shedding.

**Glossary**

**Accidental host:** An animal species that can become infected by a pathogen, but is not typically part of a pathogen's life cycle

**Reservoir host:** An animal species that carries a pathogen without detriment to itself and serves as a source of infection for transmission to new hosts

**Serovar:** A subdivision of a species or subspecies that is distinguishable from other strains on the basis of antigenic character

The challenge that lies before us now is to determine whether environmental risk assessment using quantitative leptospiral polymerase chain reaction can have an impact upon the prevalence of the infection in endemic areas. Because leptospirosis is a disease of medically underserved populations, and because data about the incidence of the disease in many areas are lacking, it will be difficult to know whether any interventional measures are effective. At least as important as molecular epidemiological methods is the need for safe and effective subunit vaccines, rapid and accurate point-of-care serodiagnostic methods, and inexpensive single-dose antibiotic therapy [6]. As with any important new public health measure, the molecular detection and identification of leptospires in environmental water samples has created new opportunities as well as new challenges.
